# Extraintestinal manifestations in ulcerative colitis patients with disease phenotype: insights from a Pakistani population

**DOI:** 10.12669/pjms.42.1.12456

**Published:** 2026-01

**Authors:** Omar Idris, Ali Akbar, Nazish Butt, Abdul Samad Bhatti

**Affiliations:** 1 Omar Idris, Department of Gastroenterology, Jinnah Postgraduate Medical Centre, Karachi, Pakistan; 2 Ali Akbar Department of Gastroenterology, Jinnah Postgraduate Medical Centre, Karachi, Pakistan; 3 Nazish Butt Department of Gastroenterology, Jinnah Postgraduate Medical Centre, Karachi, Pakistan; 4 Abdul Samad Bhatti Department of Gastroenterology, Jinnah Postgraduate Medical Centre, Karachi, Pakistan

**Keywords:** Extra-Intestinal Manifestations, Inflammatory Bowel Disease, Peripheral Arthritis, Ulcerative Colitis

## Abstract

**Background & objective:**

Ulcerative colitis (UC) is a chronic inflammatory bowel disease (IBD) involving continuous colonic mucosal inflammation that leads to extraintestinal manifestations (EIMs) in many patients. Understanding the prevalence, patterns and factors associated with EIMs is vital for optimizing UC patient care, making this study essential for assessing EIM frequency, distribution and stratification.

**Methodology:**

This observational, cross-sectional study was conducted at Jinnah Postgraduate Medical Centre in Karachi from June 2022 to November 2024, involving 147 UC patients. Prospective data collection was used to assess disease severity using the standard criteria. EIMs were classified according to their type. Statistical analysis included descriptive and inferential methods using chi-square tests with p <0.05.

**Results:**

EIMs affected 53 (36.1%) patients, with musculoskeletal manifestations in 13(8.8%) and hepatobiliary issues in 12(8.2%). Ocular involvement was observed in 8(5.4%) patients, dermatological manifestations in 09 (6.1%), renal issues (nephrolithiasis) in 5(3.4%) and systemic problems in 6(4.1%). Gender, age and disease duration did not significantly correlate with the presence of EIM (p >0.05), but severity showed a significant association (p<0.05).

**Conclusion:**

Extra-intestinal manifestations, especially musculoskeletal and hepatobiliary manifestations, are commonly found in ulcerative colitis. EIMs are correlated with disease severity and are not related to gender and age.

## INTRODUCTION

Ulcerative colitis (UC) is a chronic inflammatory bowel disease (IBD) characterized by continuous mucosal inflammation of the colon that typically extends from the rectum. It is a relapsing-remitting condition with varying disease severity and extent, classified based on the Montreal classification into proctitis, left-sided colitis and extensive colitis.^1^ Beyond gastrointestinal involvement, UC is frequently associated with extraintestinal manifestations (EIMs), affecting multiple organ systems such as the skin, joints, eyes and hepatobiliary tract^2^, which significantly contribute to disease burden and impact patient quality of life.^3^

The prevalence of EIMs in UC patients is estimated to range between 20–50%, with joint involvement, including peripheral arthritis and axial spondyloarthropathy, being the most common manifestation.^4^ Other notable EIMs include erythema nodosum, pyoderma gangrenosum, uveitis, episcleritis and primary sclerosing cholangitis (PSC), each of which may precede, coincide with, or follow the intestinal disease course.^5^ Notably, the presence and severity of EIMs appear to correlate with specific UC phenotypes, including disease extent, severity and response to therapy.^6^

Understanding the association between EIMs and UC phenotype is crucial for optimizing disease management and early intervention strategies. Patients with extensive colitis may be at a higher risk of developing multiple EIMs, while those with more severe disease activity are more likely to experience persistent or recurrent manifestations.^7^ Moreover, certain EIMs, such as PSC, have been linked to a distinct immunopathogenic profile and a higher risk of colorectal neoplasia in patients with UC.^8^ This study aimed to explore the relationship between EIMs and UC disease phenotype, highlighting potential pathophysiological mechanisms and clinical implications.

## METHODOLOGY

This cross-sectional observational study was conducted at the Jinnah Postgraduate Medical Centre (JPMC), Karachi from June 2022 to November 2024. All patients diagnosed with ulcerative colitis (UC) who attended the outpatient and inpatient departments of JPMC Karachi during this period were included and data was collected prospectively.

### Ethical approval:

It was obtained from the Institutional Review Board (IRB) of JPMC, Karachi (NO.F.2-81/2022-GENL/83/JPMC; dated: May 26, 2022) and informed consent was obtained from all participants before data collection

The sample size was determined using the WHO sample size calculator, with a 95% confidence interval, a 8% margin of error and frequency of EIMs as 42.7%, resulting in a final sample size of 147 patients.^9^

### Inclusion & Exclusion Criteria:

Patients aged 18 years and older with a confirmed diagnosis of UC based on clinical, endoscopic, histopathological and radiological findings were eligible for inclusion. To ensure diagnostic specificity, patients with a history of other inflammatory bowel diseases such as Crohn’s disease or those with incomplete medical records were excluded.

Demographic and clinical data, including age, sex, disease duration and comorbidities, were systematically recorded. Disease activity was assessed using a validated scoring system, such as the Mayo Score and categorized as mild, moderate, or severe (3-5 score for mild disease, 6-10 score for moderate disease, and 11-12 score for severe disease). The primary objective of this study was to determine the prevalence and spectrum of extra-intestinal manifestations (EIMs) in patients with UC. EIMs were classified as musculoskeletal (peripheral arthritis, spondyloarthropathy, osteopenia/osteoporosis), ocular (uveitis, episcleritis, conjunctivitis), dermatological (erythema nodosum, pyoderma gangrenosum and other skin disorders), hepatobiliary (primary sclerosing cholangitis, fatty liver disease, cirrhosis), renal (nephrolithiasis, other renal disorders) and other systemic manifestations, including thromboembolic events and oral ulcers.

Patients were systematically evaluated for these manifestations through clinical examinations, laboratory investigations, imaging studies, and skin biopsy as advised by their relevant specialties. Musculoskeletal, ocular and dermatological conditions were confirmed by referrals to rheumatology, ophthalmology and dermatology specialists. Hepatobiliary involvement was assessed using liver function tests, ultrasonography and, where necessary, liver biopsy or magnetic resonance cholangiography for confirmation of primary sclerosing cholangitis, and Doppler ultrasound of both lower limbs, hepatic veins, and portal veins, and CT pulmonary angiography after clinical evaluation of thrombosis.

The data analysis was performed using descriptive and inferential statistical methods. Continuous variables are summarized as means with standard deviations (SD), while categorical variables are presented as frequencies and percentages. Associations between EIMs and demographic or clinical variables, such as age, sex, and disease severity, were analyzed using chi-square tests for categorical variables. Statistical significance was set at P ≤0.05.

## RESULTS

This study included 147 patients as mentioned in Table-I. Most patients were female 90(61.22%) while males were 57(38.77%). A majority, 133 (90.4%) were below 50 years of age, with a mean age of 34.69±5.95. Disease duration was divided into ≤10 years 92 (62.6%) and >10 years 55(37.4%), with a mean of 8.88 years. Disease severity was mild in 52(35.4%), moderate in 46(31.3%), or severe in 49(33.3%) patients, fairly evenly distributed. The most commonly affected site of colon involvement in the study was the left side, constituting 100 (68.0%) patients.

**Table-I:** Frequency distribution of different variables (n=147).

Variables		Frequency
Gender	Male	57(38.8%)
	Female	90(61.2%)
Age groups	>50 years	14(9.5%)
	≤50 years	133(90.4%)
	Mean age (years)	34.69±15.95
Duration of disease	≤10 years	92(62.6%)
	>10 years	55(37.4%)
	Mean duration of disease (years)	8.88±5.99
Severity of disease	Mild	52(35.4%)
	Moderate	46(31.3%)
	Severe	49(33.3%)
Extent of disease	Left side colitis	100(68.0%)
	Pancolitis	45(30.6%)
	Proctitis	2(1.4%)

The frequency of extra-intestinal manifestations (EIMs) in 147 patients with UC. The overall EIM prevalence was 53(36.1%), indicating that more than one-third of the patients experienced extra-intestinal issues. Table-II. Musculoskeletal manifestations affected 13(8.8%) patients, with peripheral arthritis in 11(7.5%) and spondyloarthropathy two 2(1.4%). Dermatological issues were observed in 9(6.1%) patients, erythema nodosum in 4(2.7%) and pyoderma gangrenosum in 5(3.4%). Ocular involvement was observed in 8(5.4%) patients, uveitis in 7(4.8%) and episcleritis in 1(0.7%). Hepatobiliary conditions were observed in 12(8.2%) patients, PSC in 4(2.7%) and NAFLD in 8(5.4%). Renal problems were observed in 5(3.4%) patients with nephrolithiasis.

**Table-II:** Frequency distribution extra-intestinal manifestations (EIM).

EIM Category	Frequency (%)
Overall EIM	53(36.1%)
Musculoskeletal Manifestations	13(8.8%)
- Peripheral Arthritis	11(7.5%)
- Spondyloarthropathy	2(1.4%)
Dermatological Manifestations	9(6.1%)
- Erythema Nodosum	4(2.7%)
- Pyoderma Gangrenosum	5(3.4%)
Ocular Manifestations	8(5.4%)
- Uveitis	7(4.8%)
- Episcleritis	1(0.7%)
Hepatobiliary Manifestations	12(8.2%)
- Primary Sclerosing Cholangitis	4(2.7%)
- Non-Alcoholic Fatty Liver Disease	8(5.4%)
Renal Manifestations	5(3.4%)
- Nephrolithiasis	5(3.4%)
Systemic Manifestations	6(4.1%)
- Oral Ulcers	1(0.7%)
- Thromboembolic Events	5(3.4%)

Systemic issues were observed in 6(4.1%) patients, thromboembolic events in 5(3.4%) and oral ulcers in 1(0.7%). These results underscore the diverse EIMs in ulcerative colitis, emphasizing the importance of early detection and treatment for better outcomes. The stratification analysis revealed no significant associations between EIM prevalence and gender, age, disease duration and extent of disease, as indicated by p-values greater than 0.05, in all comparisons, but severity showed a significant association with p<0.05, with Odds increasing with disease severity, highlighting the likelihood of systemic involvement in more severe UC as mentioned in Table IV. This suggests that the occurrence of EIM in patients with UC is not strongly influenced by these variables, emphasizing the need for further research to identify other potential contributing factors.

**Table-III:** Stratification of extra-intestinal manifestations (EIM) with respect to different variables.

Variables		Extra-intestinal manifestations (EIM)		p-value
		Yes	No	
Gender	Male	22(39.0%)	35(61.0%)	0.43
	Female	29(32.3%)	61(67.7%)	
Age groups	≤50 years	49(36.8%)	84(63.2%)	0.54
	>50 years	4(28.6%)	10(71.4%)	
Duration of disease	≤10 years	34(37.0%)	58(63.0%)	0.768
	>10 years	19(34.5%)	36(65.5%)	
Severity of disease	Mild	10(19.2%)	42(80.8%)	0.001
	Moderate	16(34.8%)	30(65.2%)	
	Severe	27(55.1%)	22(44.9%)	
Extent of disease	Left sided colitis	37(37.0%)	63(63.0%)	0.557
	Pancolitis	16(35.6%)	29(64.4%)	
	Proctitis	0(0.0%)	2(100.0%)	

**Table-IV:** Odds of Extra-intestinal Manifestations Across Disease Severity in Patients with Ulcerative Colitis (n=147)

Severity	EIM Present n (%)	EIM No n (%)	OR vs Mild	95% CI
Mild	10 (19.2%)	42 (80.8%)	Reference	---------
Moderate	16 (34.8%)	30 (65.2%)	2.24	0.90-5.61
Severe	27 (55.1%)	22 (44.9%)	5.16	2.12-12.59

## DISCUSSION

This study showed that 53(36.1%) of ulcerative colitis patients with UC experienced extra-intestinal manifestations (EIMs), consistent with previous research findings that also highlight the systemic effects of the disease beyond the gut^10^, as these manifestations may indicate more severe disease so early recognition has a significant influence on patient morbidity, treatment decisions and early involvement of multidisciplinary team. Musculoskeletal manifestations were the most common EIMs, followed by hepatobiliary, dermatological, and ocular involvement. Arthritis was the top reported EIM, affecting 11(7.5%) UC patients, aligning with the literature indicating that 10% of UC cases experience arthritis.^11^ Previous studies consistently found peripheral arthritis to be more common than spondyloarthropathy.^12^

Ocular manifestations, such as uveitis in seven (4.8%) and episcleritis in one (0.7%), were reported. Previous research suggests ocular complications in 1–12% of UC patients, aligning with these findings.^13^ The higher uveitis frequency in moderate to severe disease aligns with evidence indicating systemic inflammation’s role in ocular involvement development.^14^ Uveitis carries a risk of vision loss, necessitating prompt detection and intervention to prevent complications.^15^ The results emphasize routine eye check-ups for UC patients, especially those with severe disease.

In 9(6.1%) of patients, dermatological conditions like erythema nodosum and pyoderma gangrenosum were found, aligning with previous studies on skin involvement rates in UC.^16^ Erythema nodosum, seen in 4(2.7%) of patients, is linked to disease activity and can indicate exacerbation.^17^ Pyoderma gangrenosum, occurring more often 5(3.4%), is a severe dermatological complication that may resist treatment, especially in those with active disease.^18^ UC patients with skin symptoms should undergo dermatological assessment for timely and specific interventions.

In one study, EIMs were present in 42.7% of patients. Musculoskeletal manifestations were the most common (18.4%), involving peripheral arthritis (12.7%) and spondyloarthropathy (5.7%). Dermatological EIMs occurred in 12.8% of the patients, including erythema nodosum (8.3%) and pyoderma gangrenosum (4.5%). Ocular involvement included uveitis (6.4%) and episcleritis (3.8%). Hepatobiliary manifestations affected 14.1% of patients, with primary sclerosing cholangitis (4.5%) and non-alcoholic fatty liver disease (9.6%). Renal manifestations (nephrolithiasis) were observed in 2.5% of patients, along with systemic complications, such as oral ulcers (5.1%) and thromboembolic events (3.8%).^9^ The possible variation in results in our study could be due to changes in geographical distribution and ethnical background.

Hepatobiliary involvement, mainly primary sclerosing cholangitis (PSC), occurred in 4(2.7%) patients is consistent with previous estimates in UC (2%-7% PSC prevalence).^19^ The presence of PSC in UC raises concerns as it is linked to a higher risk of colorectal cancer and liver cirrhosis, requiring regular monitoring of liver function and surveillance.^20^ Integrating hepatobiliary assessments is crucial for UC management, especially in patients with a long disease duration or abnormal liver enzyme levels. Renal issues were rare, with nephrolithiasis affecting 5(3.4%) patients. Chronic inflammation, gut microbiota changes and increased oxalate absorption elevate the risk of kidney stones in patients with IBD, despite the low prevalence in UC.^21^

Renal involvement in UC requires monitoring, despite being less researched. Holistic care beyond GI symptoms is crucial to effectively managing daily functioning. Sex differences in EIM prevalence are minimal: males show slightly more musculoskeletal issues and females tend to have skin problems, aligning with past findings of subtle sex-based variations.^22^

In our study, patients with severe disease manifestations were over five-fold as likely to develop extraintestinal manifestations, with an odds ratio of 5.16, in comparison to those with mild disease. This observation is in line with the multinational study^23^, indicating that greater intestinal inflammation and systemic immune activation in severe disease increase the risk of extraintestinal manifestation. This may be possibly explained by the pathophysiological mechanism, whereby the immune cells are activated due to inflammation, allowing gut-primed lymphocytes cytokines to target joints, skin, and eyes. Furthermore, gut dysbiosis along with increased permeability exposes the immune cells to microbial antigens, promoting cross-reactivity. Common genetic roots and overlapping immune pathways, including IL-23/Th17 and TNF, connect intestinal and extraintestinal manifestations. This “spillover” of immune activity explains why greater disease severity correlates with more extraintestinal manifestations (EIMs).^24^

### Limitations

This study highlights the prevalence and characteristics of EIM in UC but has limitations: a single-center, cross-sectional design inherently limits the ability to capture intermittent or transient extra-intestinal manifestations (EIMs). Limited access to diagnostic and multidisciplinary healthcare facilities may have led to underdetection of extraintestinal manifestations.

## CONCLUSION

Extra-intestinal manifestations, especially musculoskeletal and hepatobiliary manifestations, are commonly found in ulcerative colitis. EIMs are correlated with disease severity and are not related to gender and age.

### Recommendations

Future studies should use multicenter designs with standardized criteria to ensure better reliability. Longitudinal studies are needed to understand the progression of EIMs and their response to treatment. Comprehensive care involving various specialties is crucial for optimal outcomes. Vigilant monitoring is the key to clinical practice.

### Author’s Contribution:

**OI NB:** Conceived and designed the study, interpretation of data, responsible for accuracy and integrity of study.

**AA:** Conceived and designed the study, interpretation of data, critical review, and final approval for publication.

**ASB:** Collection, interpretation, of data, performed statistical analysis, critical review.
